# The effects of beta acids from hops (*Humulus lupulus*) on mortality of *Varroa destructor* (Acari: Varroidae)

**DOI:** 10.1007/s10493-012-9593-2

**Published:** 2012-07-06

**Authors:** Gloria DeGrandi-Hoffman, Fabiana Ahumada, Gene Probasco, Lloyd Schantz

**Affiliations:** 1 Carl Hayden Bee Research Center, USDA-ARS, 2000 East Allen Road, Tucson, AZ 85718 USA; 2 BetaTec Hop Products, Division of John I. Haas Inc., 5185 MacArthur Blvd, N1 V, Suite 300, Washington, DC 20016 USA

**Keywords:** *Varroa destructor*, *Apis mellifera*, *Humulus lupulus*, Package bees

## Abstract

Hop (*Humulus lupulus* L.) beta acids (HBA) were tested for miticidal effects on *varroa destructor* Anderson and Trueman, a parasitic mite of the honey bee (*Apis* *mellifera* L.). When varroa were placed on bees that had topical applications of 1 % HBA, there was 100 % mite mortality. Bee mortality was unaffected. Cardboard strips saturated with HBA and placed in colonies resulted in mite drop that was significantly greater than in untreated hives. HBA was detected on about 60 % of the bees in colonies during the first 48 h after application. Mite drop in colonies lasted for about 7 days with the highest drop occurring in the first 2–3 days after treatment. There was a reduction in the percentages of bees with HBA and in the amounts on their bodies after 7 days. Bee and queen mortality in the colonies were not affected by HBA treatments. When cardboard strips saturated with HBA were put in packages of bees, more than 90 % of the mites were killed without an increase in bee mortality. HBA might have potential to control varroa when establishing colonies from packages or during broodless periods.

## Introduction

The varroa mite (*Varroa destuctor* Anderson and Trueman) is a major pest of honey bees (*Apis mellifera* L.) that has caused colony losses throughout the world (De Jong et al. 1982; Rosenkranz et al. 2010). Varroa is an ectoparasite that feeds on developing brood and adults. Colonies infested with varroa usually die within 2–3 years if left untreated. Varroa harm bees by parasitizing worker and drone brood causing a shortening of adult lifespans (Rosenkranz et al. 2010). Short-lived adults impact the demographics of the colony population and over time can cause colonies to perish (DeGrandi-Hoffman and Curry 2004). Varroa also transmit many types of virus during feeding causing further harm to colonies (Ball and Allen 1988; Bowen-Walker and Gunn 1998; Bowen-Walker et al. 1999; Chen et al. 2004; Shen et al. 2005; Di Prisco et al. 2011).

Varroa populations increase in hives during periods when the colony population is growing. Mated female mites (foundress) invade brood cells just before they are capped for pupation (Martin 1994). Male and female offspring are produced and mate under the sealed cell. The mated female mites leave the cell when the bee emerges and in this phoretic stage search for new cells to infest (Sammataro et al. 2000). It is during this phoretic stage that the mite is most vulnerable to control measures.

There are several commercially available products to control varroa. These include plastic strips impregnated with tau-fluvalinate (Apistan®, Wellmark International, Bensenville, IL) or coumaphos (CheckMite-+, Bayer, Shawneee Mission, KS, USA). Formic acid (MiteGone, MiteGone Enterprises International, Kelowna, British Columbia, Canada or MiteAway Quick Strips, NOD Apiary Products, Frankford, Ontario, Canada), Thymol (e.g., Apiguard, Vita (Europe) Limited, Valdosta, GA, USA) and other plant essential oils also are available. Oxalic acid also has been used to control varroa in Europe and Canada (Gregorc and Planinc 2001). Though each of these control methods can be effective in reducing varroa populations, they also have limitations. In the US and Europe, varroa have become resistant to fluvalinate (Elzen et al. 1999; Milani 1999; Johnson et al. 2010) and coumaphos (Spreafico et al. 2001; Elzen and Westervelt 2002; Pettis and Jadczak 2005; Sammataro et al. 2005) so their effectiveness has been reduced. Additionally, fluvalinate and coumaphos are lipophilic and contaminate the wax comb where bees store food and rear brood (Cabras et al. 1997; Wallner 1999; Mullin et al. 2010). Formic acid can effectively reduce varroa populations but control is dependent on ambient temperature. Under cool conditions formic acid can be ineffective while under high temperature it can harm adult bees and brood (Elzen et al. 2004) and cause queen loss (Giovenazzo and Dubreuil 2011). Thymol and oxalic acid have been used successfully to control varroa, but efficacy is influenced by temperature, humidity (Skinner et al. 2001), brood area (Eischen 1998) and colony size (Emsen and Dodologlu 2011). There are studies where oxalic acid was effective in reducing mite populations (Toomemaa et al. 2010) and where it was not (Emsen and Dodologlu 2011). Plant essential oils also have been tested for varroa control (Sammataro et al. 1998; Damiani et al. 2009) but difficulty in developing consistent delivery methods, comb and honey contamination and their toxicity to bees have limited their use.

Compounds that have not been previously considered for controlling varroa are hop beta acids (HBA). These compounds (lupulones) are naturally occurring weak organic acids produced by hop plants (*Humulus lupulus* L.) (Jones et al. 2003). The compounds repel sucking plant pests including two-spotted spider mite (*Tetranychus urticae* Koch) (Jones et al. 1996) and hop aphid (*Phorodon humuli* Schrank) (Hampton et al. 2002; Jones et al. 2003). HBA also can reduce two-spotted spider mite oviposition and reduce the survival of adults (Jones et al. 1996).

Because HBA from hops are readily available and non-toxic to humans, we tested them for miticidal activity on varroa mites. To adequately control varroa in honey bee colonies, the compounds would need to get onto the bodies of worker bees and be spread throughout the colony population at levels that do not harm the bees but repel or kill phoretic varroa. The objectives of the studies were to determine the feasibility of using HBA to control varroa. We first exposed HBA at different concentrations directly to bees to test for toxicity. We measured varroa mortality to those concentrations of HBA that did not cause bee mortality. We tested the effectiveness of HBA in reducing mite populations in colonies and in packaged bees by applying HBA on cardboard strips and measuring mite drop. We also measured the amounts of HBA on the bodies of the bees in packages and in colonies over time to determine how long the compound remained in an active state. Results from our studies were used to determine how HBA might be best used to control varroa mites.

## Materials and methods

The HBA used in this study were provided by BetaTec Hop Products (Washington, DC, USA). The HBA were extracted from the cones of hop plants.

### HBA toxicity to bees

Toxicity of HBA to worker bees was evaluated by topically applying 0.5 μl solutions of HBA diluted in propylene glycol (PG) to the abdomen of 1 day-old worker bees. Concentrations of 0.5, 1.5, 2.0, 4.0, and 9.0 % HBA were tested. For controls, we applied 0.5 μl of just PG per bee. Each treatment concentration and control was applied to five bees. After treatment, the bees were placed in a petri dish and kept in an incubator at 34 °C and 20–30 % RH. The procedure was replicated in three petri dishes for each HBA concentration and for controls. Mortality was observed hourly for the first 5 h and then at 21 h. These times were chosen because pilot tests with different concentrations of HBA indicated that the highest bee mortality occurred during the first 5 h after exposure to HBA especially at the higher HBA concentrations. Mortality did not increase greatly between 5 and 21 h or afterwards.

**Fig. 1:**
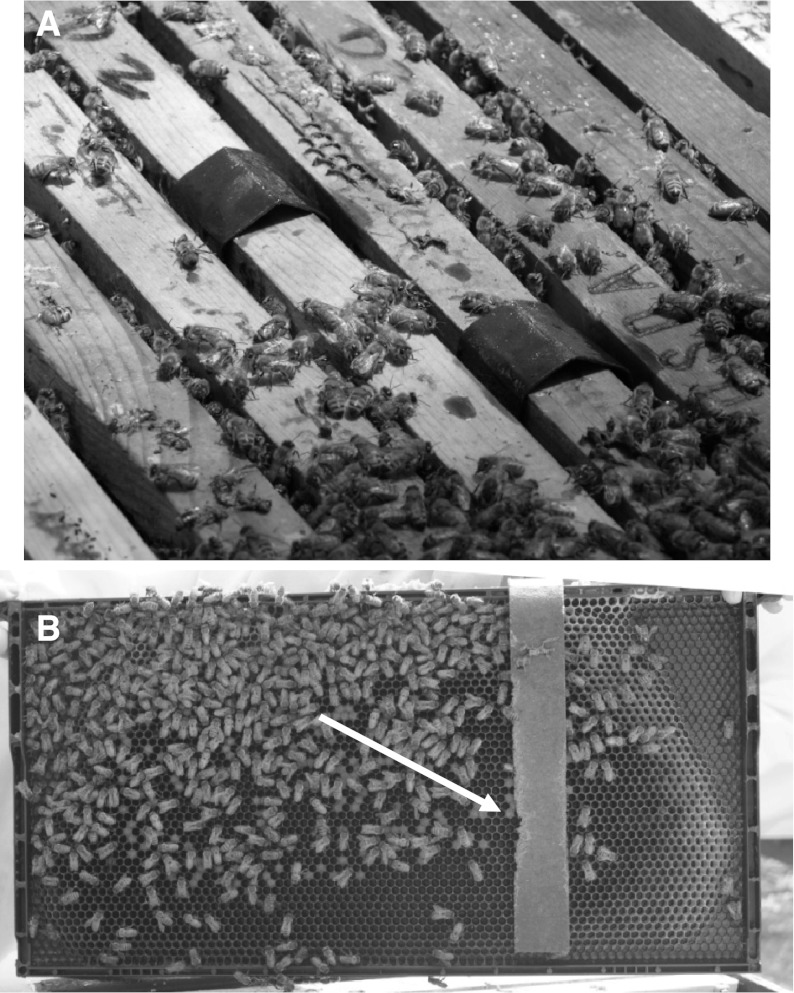
Card board strips saturated with hop beta acids placed between frames in a honey bee colony (**a**). The strip hanging down on to the frame and indications of where it was chewed on by the bees (*arrow*) are shown in **b**

The effect of HBA on honey bees in colonies was determined at the Carl Hayden Bee Research Center (CHBRC) apiary in Tucson, AZ, USA. Two cardboard strips (44.4-cm × 3.2-cm) with 3.84 g of a 16 % HBA solution suspended in a PG and polysorbate 60 solution (hereafter referred to as HBA strip) were placed between the center frames in European honey bee colonies (Fig. 1). Treatment and control colonies were composed of two standard deep Langstroth hive boxes with an average of 14.3 ± 0.7 frames of bees and brood. Ten colonies each were used for the treatments and controls. The colonies were equipped with dead bee traps to determine the daily number of bees dying in colonies. The traps were placed on the colonies 96 h prior to adding the treatments to obtain pre-treatment counts of dead bees. The dead bee traps were checked every 24 h for 96 h after the strips were inserted.

Mortality from HBA also was determined in commercial packaged bees. The packaged bees were obtained from Steve Park Apiaries, Palo Cedro, CA, USA. The bee packages were assembled in Palo Cedro, CA and then transported by ground to the CHBRC. Packages had 0.9 or 1.4 kg of bees (about 6700 and 9000 bees respectively), a caged queen and a container of sugar candy. The packages were equipped with removable sticky boards covered with 0.32-cm^2^ mesh hardware cloth attached to the bottom of the package so that mite mortality also could be evaluated (see below). HBA strips used in packages were one-half the size of those used in colonies (i.e., 22.2-cm × 3.2-cm) and were saturated with a 16 % AI solution of HBA (1.92 g of HBA per strip). The strips (hereafter referred to as HBA package strips) were hung inside the package prior to transport. Two, three, or four package strips were attached to the top inside area of the package on either side of the queen cage. Five packages were used for each treatment. An additional five packages of bees received no HBA strips (controls). Dead bees were counted 48 h after treatment. The total number of bees in each package was counted to determine the percentage mortality.

### Varroa mortality

Concentrations of HBA that caused little or no bee mortality (0.5 and 1.0 %) were applied to 1 day-old worker bees to determine the effects on varroa. A 0.5 μl droplet with HBA diluted in PG was applied topically to the abdomen. The droplet delivered 500 μg of either a 0.5 or 1 % solution. Therefore, either 2.5 or 5 μg of HBA was applied per bee, respectively. Following the application, a live mite was placed on the treated bee. The mites had been captured in the phoretic stage from colonies. As controls, 0.5 μl of PG alone was topically applied to the abdomen of bees. Five bees were used for each treatment and were placed in a petri dish and kept in an incubator at 34 °C and 20–30 % RH. Mite mortality was recorded hourly for 5 h and then at 21 h after treatment.

The efficacy of HBA in reducing varroa populations in colonies was tested in 5-frame hives with wooden bottom boards at the CHBRC apiary in September, 2010. Each colony contained 4,000–5,000 bees and a laying queen. Seven colonies were used for each treatment and an additional 7 colonies for controls. So that all the mites in the colonies would be exposed to the treatments, we interrupted brood production by confining the queen in an 8.6 cm × 5.4 cm wire mesh hardware cloth cage (30 gauge) on the day that the HBA treatments were placed in colonies. The queens remained in the cages for 13 days. This allowed time for all mites in the colony to emerge from the sealed brood and be exposed to the HBA strips. Having the queen caged for 13 days also insured that there was not any new brood of suitable age for infestation during the 21 day experimental period.

The effects of HBA strips on mite populations in colonies were evaluated using two different treatment schedules. For Treatment A, 2 HBA strips were placed in colonies for 21 days. In Treatment B, 2 HBA strips were placed in colonies, and then replaced with two new strips 14 days later. Control colonies received no HBA treatments. Sticky boards were placed on the bottom boards of all colonies on the day the strips were inserted. Mite drop was counted 48 h later. The strips were removed from all treatment colonies on day-21, and one Apistan® (fluvalinate) strip was inserted in each colony. Sticky boards were placed on the bottom boards to estimate mite drop. Apistan® strips were removed 4 days later and the mites on the sticky boards were counted. One week after the Apistan® treatment, two HBA strips were inserted in all colonies, and a sticky board was inserted on the bottom board. Boards were left in the colonies for 48 h, and the number of mites that dropped was counted.

The effect of HBA strips on mites in package bees was determined using the methods described above for the effects on bee mortality. Mite mortality in packages was evaluated at Pendell Apiaries, Stonyford, CA (site-A) and Steve Parks Apiaries (site-B). In all cases, 5 packages were used per treatment and control. At site-A, we tested HBA strips in 0.9 kg packages, and at site B in 0.9 and 1.4 kg packages. Mortality was measured by counting the mites that dropped on to the sticky boards placed on the bottom of the packages. To determine the number of mites still remaining on the bees, we put the packages in a freezer until the bees were inactive. Then, the bees were submerged in 70 % ethanol to dislodge the remaining mites. The bees were submerged in a 3.8-l plastic canister containing the ethanol, shaken for 1 min and then placed in a double strainer. The top strainer contained the bees and mites. The bottom strainer had a paper towel on the bottom to catch the mites that were washed off the bees. The number of mites on the paper towel was recorded. The bees were then separated into 4 groups of approximately equal size and the alcohol wash was repeated three times with each group. The total number of mites washed from the bees was recorded. The number of mites on the sticky boards was added to the number counted from the alcohol washes to determine the total number of mites for the package. The total number of mites counted on sticky boards in each package was divided by the total number of mites counted for each package (sticky board counts + mites from alcohol washes) to determine the proportion of mites killed after the 48 h treatment period by the HBA strips.

### Quantitative analysis of HBA on bees

The amount of HBA on bees was determined in the studies to test efficacy in colonies and in packages. Bees were sampled from the center frame where the strip was hanging. Ten bees were collected per sample and placed in individual Eppendorf tubes. Bees were frozen (−80 °C) until analysis for HBA. Bees were sampled 2, 7, 16, and 21 days after the strips were placed in the colonies. Three bees out of the 10 sampled per colony were used to estimate the average amount of HBA per bee. In the studies evaluating HBA strips in packaged bees, the amount of compound on the bees was determined by collecting 30 bees per package and analyzing the pooled sample. Bees in packages were sampled before performing the alcohol wash.

The analysis to determine amounts of HBA on bees was conducted by Beta Tec Hops Products. Prior to the analysis, a standard solution of HBA was made by weighing 0.4000–0.6000 g (±0.0001 g) of standard hop extract and adding it to 50 ml of high-pressure liquid chromatography (HPLC) grade methanol in a volumetric flask. The flask was sonicated (Branson 1510 sonicator) to dissolve the extract. One milliliter of stock solution was added to 9.0 ml of HPLC grade methanol. The standard solution was kept frozen until used for HPLC calibration. Four standard injections were run each time a bee sample analyses was performed. The standard deviation of the four injections was equal to or less than a 2 % Relative Standard Deviation (RSD).

Individual bees were placed in centrifuge tubes containing 1 ml of a 50/50 mixture of HPLC methanol and toluene. The bees were soaked in the solution for 20 min. The solvent was then placed in HPLC vials for analysis. The samples were analyzed on an Agilent 1100 HPLC with a UV detector and a Nuclosil C18 5 μ, 4.6 × 250 100 Å column. The HPLC column temperature was set to 40 °C, pump flow rate 1.00–1.20 ml/min, UV detector 350 and 314 nm and set injector to 40 μl. A The HPLC was run on a 350 nm wavelength method with a 40 μl sample injection, a 2 μl standard injection and a blank. The HPLC chromatograms provided individual areas for the HBA components, co-lupulone and lupulone, which were summed to calculate the total beta area (β area = CLup + Lup). The total areas, along with concentrations and injection volumes, were compared to those of the calibration extract using the equation:

$$ \mu{\text{g}}\beta /{\text{Bee}} = \frac{{{\text{Sample}}\,\beta \,{\text{area}}}}{{{\text{Average standard }}\beta \,{\text{area}}}} \times \frac{{\mu {\text{g}}/{\text{ml\,standard}}}}{{{\text{Bee}}/{\text{ml\,sample}}}} \times \frac{{\mu {\text{l\,injection\,standard}}}}{{\mu {\text{l\,injection\,sample}}}} $$

### Statistical analysis

The number of bees counted in dead bee traps after applying HBA strips was compared with pre-treatment values for each sampling interval using a two-way analysis of variance (ANOVA) with presence of HBA strips and time after application as effects. Bee mortality in packages was compared using a two-way ANOVA using number of strips and size of package as factors. One-way ANOVA was conducted separately for each size package to compare bee mortality among treatments and control. Mite mortality in colonies (expressed as a proportional increase in mite drop relative to pre-treatment counts) was compared among treatments with a two-way ANOVA using treatment type and sample day as effects. The average number of mites counted on sticky boards 48 h after adding HBA strips to colonies was compared between treatments and controls using a one-way ANOVA. At the conclusion of the study, the total number of mites counted on the sticky boards was summed and used as an estimate of the mite load in the colonies. The estimated number of mites per colony was averaged for each treatment group and a one-way ANOVA was conducted to compare average mite loads across treatment groups. Comparisons of mite mortality in package bees were made using a one-way ANOVA with number of strips as the fixed factor. Separate analyses were conducted for each size of package. The percentage of bees in treatment A and B colonies where we could detect HBA was compared using a *t* test. The average amount of HBA detected on bees in treatment A and B colonies over time was compared using a two-way ANOVA with treatment type and time as effects in the analysis. The average amount of HBA per bee in packages was estimated based on a pooled sample of 30 bees per package. Comparisons of HBA per bee were made among packages with different numbers of strips using a one-way ANOVA. Separate analyses were made for each size of package. A two-way ANOVA was conducted on average amounts of HBA per bee using number of strips and size of package as fixed factors.

## Results

### HBA toxicity to bees

**Fig. 2:**
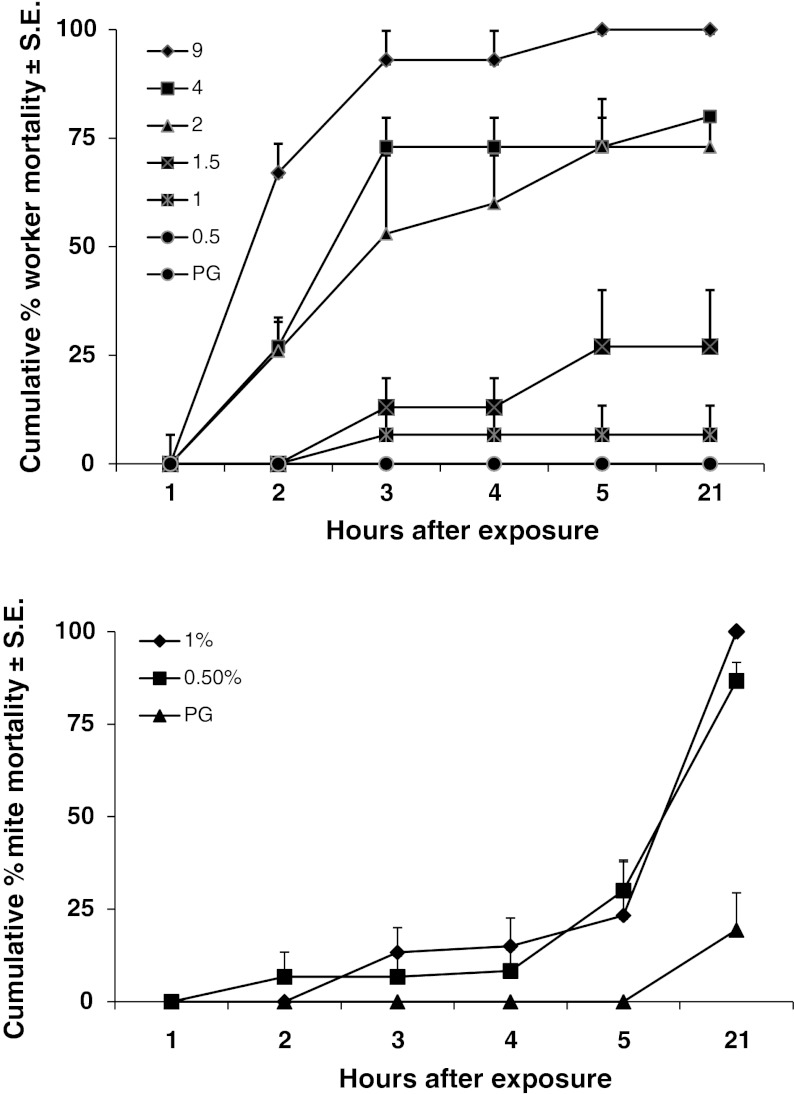
Worker honey bee and *Varroa* mite mortality after topical applications (0.5 μl per bee) of hop beta acids diluted in propylene glycol (PG) on to the abdomen of the bee

Applying 0.5 μl of HBA at 9 % concentration on to the abdomens of worker bees resulted in 100 % mortality during a 21 h period (Fig. 2). Mortality dropped with decreasing concentration. At 1 % concentration, bee mortality was less than 5 % after 21 h. There was no bee mortality when 0.5 % concentration or PG alone was applied to bees.

When HBA strips were placed in colonies, there was no significant increase in bee mortality during any sampling interval (*F*_3,72_ = 0.09, *p* = 0.96) (Table 1). The average number of bees counted on dead bee traps during the 96 h pre-treatment interval was 6.9 ± 3.3 bees per colony. During the 72 h post-treatment interval, mortality averaged 8.2 ± 1.5 bees per colony. These averages were not significantly different (*F*_1,72_ = 0.12, *p* = 0.73). Similarly, control colonies did not differ in bee mortality during the pre- and post-treatment interval (*F*_1,72_ = 0.13, *p* = 0.71).

**Table 1:** Two-way ANOVA of changes in bee mortality (bee mortality post treatment/bee mortality pre-treatment) after hop beta acid (HBA) saturated strips were placed in their colonies. Mortality was recorded at 24 h intervals (referred to as ‘sample time’) for 96 h before (pre-treatment) and 96 h after treatment (post-treatment). Mortality was recorded in a similar manner during the study period in control colonies that did not have HBA strips

Factor	Degrees of freedom	MS	*F*	*p*
Control colonies				
Pre- versus post treatment	1	27.613	0.12	0.73
Sample time	3	20.879	0.09	0.96
Interaction	3	33.913	0.15	0.93
Error	72	223.882		
Treatment colonies with HBA strips				
Pre- versus post treatment	1	35.112	0.13	0.71
Sample time	3	271.513	1.04	0.38
Interaction	3	227.379	0.87	0.46
Error	72	260.279		

Tests of toxicity of HBA strips to bees in packages indicated no significant difference in bee mortality between 0.9 kg packages with strips and controls (*F*_3,16_ = 2.22, *p* = 0.13). Mortality with 2 strips was 1.06 ± 0.13 %, 3 strips was 1.2 ± 0.2 % and 4 strips was 0.96 ± 0.37 %. There was 0.44 ± 0.9 % mortality in 0.9 kg packages without strips. In the 1.4 kg packages, worker mortality was significantly higher in packages with 4 strips compared with those with 2 or 3 strips or controls (*F*_3,16_ = 5.18, *p* = 0.011). Mortality was 2.7 ± 0.4 % in packages with 4 strips compared with 1.0 ± 0.6 % and 1.4 ± 0.3 % in packages with 2 or 3 strips, respectively. Mortality in packages without strips was 0.62 ± 0.12 %.

### Mite mortality from HBA

Applying 0.5 μl of either a 0.5 or 1.0 % concentration of HBA diluted in PG to the abdomen of bees resulted in mite mortality of 30 and 23 % respectively after the first 5 h of exposure (Fig. 2). Some mites crawled off the bees, and we interpreted this as repellency. There was no mite mortality and none of the mites crawled off the bees treated with PG alone. After 21 h, 100 % of the mites treated with 1 % HBA and 86.7 % of those treated with 0.5 % were dead. In controls with PG alone, 19 % of the mites died after 21 h.

**Fig. 3:**
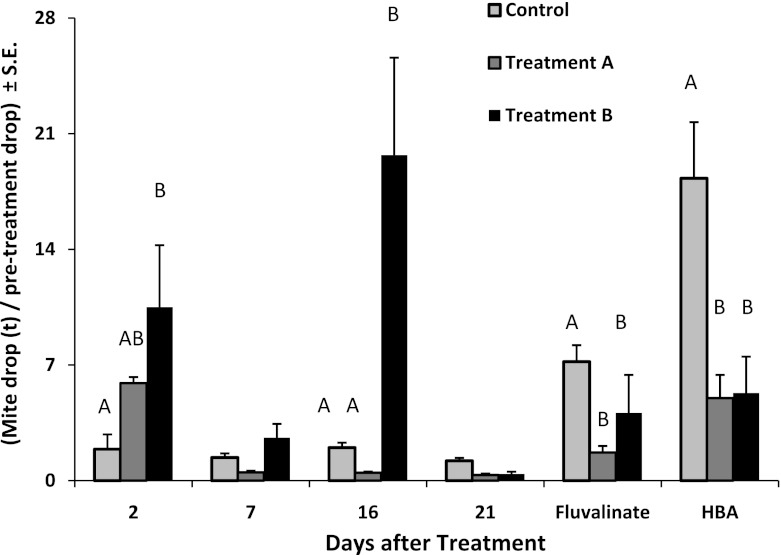
Change in varroa mite drop (number of mites on sticky boards on days after treatment/mites counted prior to treatment) on days following a single application of hop beta acid saturated strips in colonies (Treatment A) or when the single application was followed by a second application 14 days later (Treatment B). Control colonies received no treatments. After 21 days, all colonies were treated with fluvalinate. A week later, the change in mite drop was measured 2 days after application of hop beta acid (HBA) saturated strips. Means followed by the same letter for each exposure interval are not significantly different as determined by a Tukey’s W test

In colonies, the average number of mites counted on sticky boards prior to treatments with HBA strips was: 40.6 ± 21.1 for treatment A, 25.3 ± 8.2 for treatment B and 13.7 00B1 1.4 for control colonies. These averages were not significantly different (*F*_2,18_ = 1.06, *p* = 0.37). The proportional change in mite drop relative to the pre-treatment counts in colonies where HBA strips were inserted differed among treatment groups (*F*_2,124_ = 7.47, *p* = 0.001), sampling days (*F*_5,124_ = 7.85, *p* < 0.0001), and in the interaction between the two factors (*F*_10,124_ = 7.83, *p* < 0.0001) (Fig. 3). The highest mite drops relative to pre-treatment counts occurred in treatment colonies during the first 2 days after HBA strips were placed in colonies (*F*_2,18_ = 3.74, *p* = 0.04). The average number of mites counted on sticky boards 2 days after the first HBA treatment was 249 ± 134 for treatment A, 267 ± 153 for treatment B and 23.4 ± 9.4 for controls. The average mite drop did not differ between treatment A and B colonies but both was significantly higher than controls (*F*_2,18_ = 9.92, *p* = 0.001). By day 7, mite drop did not differ among treatments and controls (*F*_2,18_ = 0.75, *p* = 0.49). Mite drop in Treatment-B was significantly higher than Treatment A or controls 2 days after new HBA strips were put into colonies (day 16) (*F*_2,18_ = 9.83, *p* = 0.001). By day 21, significantly more mites were counted on sticky boards in control colonies compared with either Treatment A or B (*F*_2,18_ = 10.75, *p* = 0.001).

During the entire study period, the total number of mites counted on sticky boards averaged 436 ± 62.6 for control colonies, 582 ± 300 for colonies in treatment A and 1,017 ± 483 for treatment B. These averages did not differ significantly (*F*_2,17_ = 0.9, *p* = 0.46). During the first 21 days of the study, 20.7 ± 0.03 % of all the mites that dropped on to sticky boards during the entire study period were counted in control colonies while 51 ± 0.04 and 75.6 ± 0.04 % were counted in treatments A and B, respectively.

When Apistan® strips were inserted on day-21, there was no significant difference in mite drop relative to pre-treatment counts among treatment or control colonies (*F*_2,18_ = 3.54, *p* = 0.051). However, when HBA strips were inserted 1 week later, significantly more mites were counted on sticky boards in control colonies than in either treatment groups (*F*_2,18_ = 22.06, *p* < 0.0001). The percentage of total mites counted on sticky boards following treatments with HBA strips (during both the 21 day interval after initial treatments and after the final HBA treatment) averaged 81.3 ± 0.3 % for treatment A and 88.2 ± 0.3 % for treatment B. The single application of HBA strips to control colonies resulted in mite drop representing an average of 52.4 ± 0.6 % of all the mites counted.

**Fig. 4:**
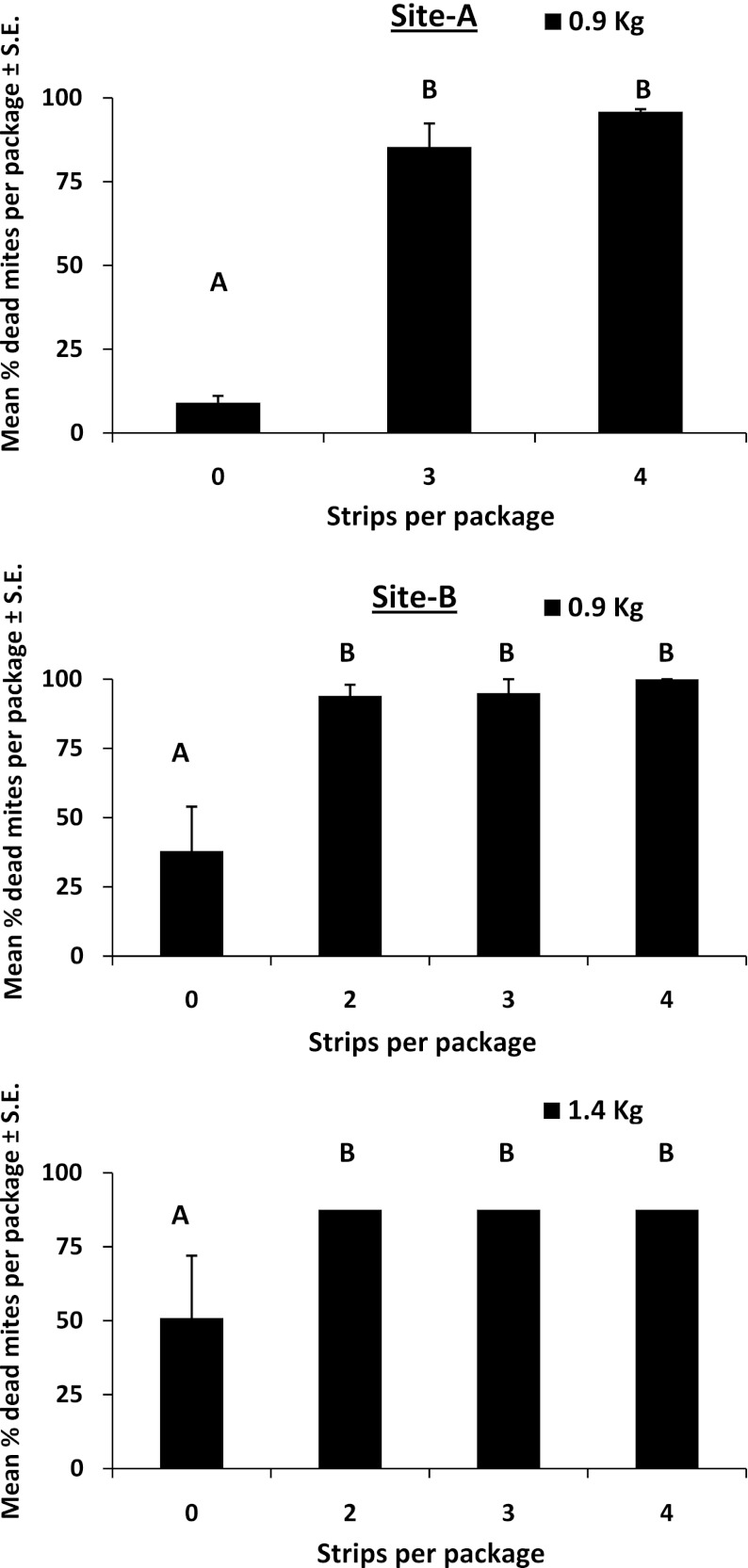
Average *Varroa* mite mortality in package bees weighing 0.9 or 1.4 kg and containing 0–4 strips saturated with hop beta acids. Means followed by the same letter are not significantly different at the 0.05 level as determined by a one-way analysis of variance followed by a Fisher multiple comparison test. Test statistics for site A are: *F*_2,17_ = 102.5, *p* < 0.0001, site B 0.9 kg: *F*_3,16_ = 11.38, *p* < 0.0001, site B 1.4 kg: *F*_3,16_ = 4.48, *p* = 0.18

Packages of bees from site A had between 61–269 mites per 0.9 kg packages. When HBA strips were placed in the packages, there was significantly greater mite mortality (85–96 %) than in controls (9.1 %) (*F*_2,17_ = 102.5, *p* < 0.0001) (Fig. 4). Mortality did not differ between packages with 3 or 4 strips. On average, 169 ± 22.3 mites were counted on sticky boards on the bottom of the packages with 3 HBA strips and 207 ± 15.2 with 4 HBA strips. Control packages had an average of 8.2 ± 1.2 mites on the sticky board. At site-B, there were 1–13 mites per 0.9 kg package and 5–20 in the 1.4 kg. Mite mortality from strips placed in 0.9 kg packages ranged from 94–100 % (average number of mites per sticky board = 7.2 ± 2.0, 6.2 ± 1.4, and 7.2 ± 1.5 for 2, 3, and 4 strips respectively). Packages with HBA strips had greater mortality than controls (*F*_3,16_ = 11.38, *p* < 0.0001). Mite mortality with 3 or 4 strips did not differ from those with only 2 strips (Fig. 4). In 1.4 kg packages, mite mortality ranged between 94–99 % (average number of mites on sticky boards = 7.6 ± 1.4, 10.2 ± 1.2, and 14.2 ± 1.4 for 2, 3, and 4 strips respectively). There was no significant difference in mite mortality with increasing numbers of strips. Packages with strips had significantly greater mortality than controls (*F*_3,16_ = 4.48, *p* = 0.18; average = 2.0 ± 0.84 mites on the sticky board).

### HBA on bees

**Fig. 5:**
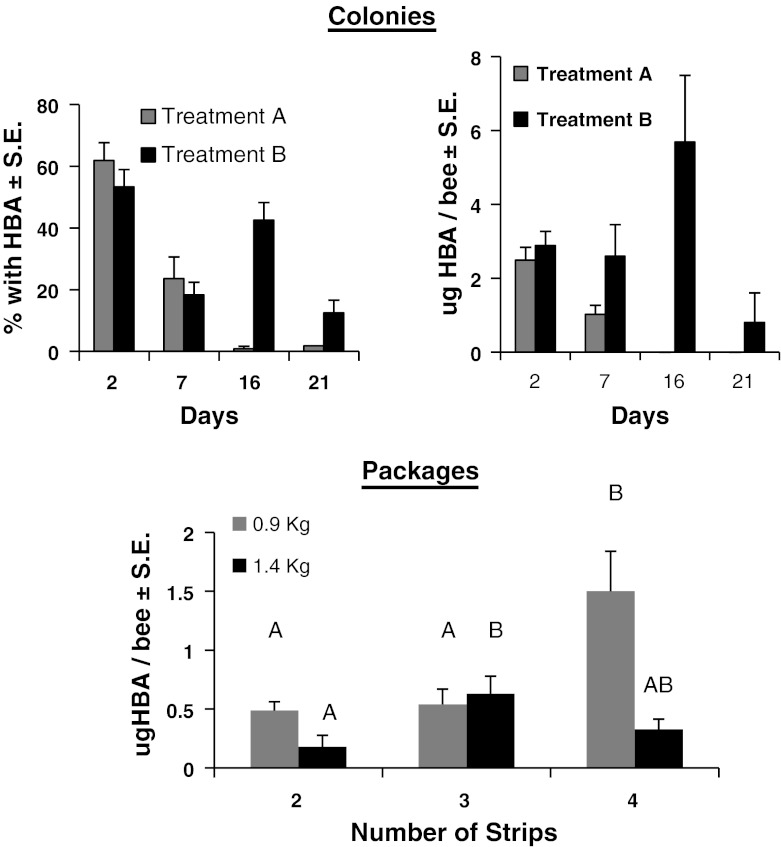
Percentage of worker honey bees with detectable levels of hop beta acids (HBA) and average amounts of HBA detected on bees in colonies on days following treatment with strips saturated with HBA. In colonies, measurements were made after either a single application (Treatment A) or with a second application 14 days later (Treatment B). Means are not shown for Treatment A on days 16 or 21 because HBA was detected on 1 % of bees sampled on day-16 and on only one bee from two colonies on day-21. Measurements of HBA were made in packages 2 days after application. Average amounts of HBA followed by the same letter are not significantly different as determined by a Student’s *t* test (*p* < 0.05)

We tested for the presence of HBA on bees 2 days after strips were placed in colonies. The compound was detected on 61.8 ± 5.8 % of the bees in Treatment A and 53.3 ± 5.6 % in Treatment B (Fig. 5). These averages did not differ between the treatments (t = 1.05, d.f. = 20, *p* = 0.31). We did not detect HBA in any of the control colonies. The average amount of HBA per bee 2 days after the HBA strips were applied did not differ among colonies in either treatment (Treatment A: *F*_10,32_ = 0.58, *p* = 0.80; treatment B: *F*_11,35_ = 0.82, *p* = 0.52). Consequently, the values for all colonies within a treatment group were combined. A *t* test indicated no significant difference in the amount of HBA per bee between treatment groups (t_66_ = 0.34, *p* = 0.73) (Fig. 5). The average amount of HBA per bee was 2.49 ± 0.53 μg in Treatment A colonies and 2.89 ± 0.6 μg in Treatment B.

After the strips were in the colonies for 7 days, about 24 % of the bees from Treatment A had detectable levels of HBA and 18.3 % of those from treatment B. On the bees where we could detect HBA, the average amount per bee did not differ among colonies within each treatment group (Treatment A: *F*_6,12_ = 0.8, *p* = 0.59; Treatment-B: *F*_11,24_ = 0.84, *p* = 0.61), so colony values were combined for each treatment. The average amount of HBA on bees 7 days after strips were placed in colonies did not differ between treatments (Treatment A = 1.03 ± 0.24 μg; Treatment B = 2.6 ± 0.85 μg) (t_40_ = 1.79, *p* = 0.081).

Two weeks after the first strips were put into colonies; additional strips were added to Treatment B colonies. We detected HBA 2 days after application on about 42 % of the bees from Treatment-B. Only about 1 % of the bees in Treatment-A colonies had detectable levels of HBA 16 days after the initial application. The percentage of bees with HBA in treatment B colonies was significantly higher than in treatment A (t_11_ = 7.29, *p* < 0.0001). The percentage of bees with HBA in Treatment B after the second application of HBA strips did not differ from the percentage after the first application (t_21_ = 1.37, *p* = 0.19). The average amount of HBA on bees in treatment B after the second application was 5.69 ± 1.8 μg/bee. This was not significantly different from average amounts of HBA on bees 2 days after the first application of HBA strips (t_38_ = 1.49, *p* = 0.14).

Twenty-one days after the start of the study, we detected HBA on bees in only two of treatment A colonies we sampled. Those colonies had only one bee that tested positive. The average amount of HBA on the bees was 0.805 μl/bee; n = 2. In Treatment B, 58 % of the colonies had at least one bee that tested positive for HBA; the average amount per bee was 1.44 ± 0.267 μg; n = 7. The mean amount of HBA per bee for day 21 was estimated using a single random sample from each colony where we detected HBA on at least one bee.

The average amount of HBA detected on bees in packages was significantly affected by the size of the package (*F*_1,24_ = 10.50, *p* = 0.003), the number of strips used (*F*_2,24_ = 5.99, *p* = 0.008) and the interaction between them (*F*_2,24_ = 7.05, *p* = 0.004). There was significantly more HBA on bees from 0.9 kg packages with 4 strips compared with those having 2 or 3 (Fig. 5). In the 1.4 kg packages, bees had significantly more HBA on their bodies when 3 strips were used compared with 2 strips. Packages with 4 strips did not differ from those with 2 or 3 strips. We did not detect HBA on bees in control packages.

## Discussion

HBA from hops plants are miticidal and effective feeding and oviposition deterrents of certain phytophagous mites (Jones et al. 1996, 2003). Our studies indicate that these compounds also can cause mortality in varroa mites. The miticidal activity of HBA was demonstrated by exposing varroa directly to the compound and measuring mortality. Amounts of HBA that were lethal to mites did not cause significant mortality to the bees or their colonies. Sufficient amounts of HBA to cause mite mortality could be delivered on to bees in colonies or packages by inserting cardboard strips saturated with HBA. The highest varroa mortality from HBA was in packaged bees. In colonies, HBA increased varroa mortality, but the effect lasted for only a week. Multiple applications would be needed to reduce varroa populations in highly infested colonies with capped brood. Mite drop from HBA in colonies was greater than with fluvalinate indicating the presence of resistant mites and their susceptibility to HBA.

Amounts of HBA required to kill varroa as determined by the petri dish bioassays were detected within 2 days of inserting the HBA strips into either colonies or packages. HBA probably was dispersed among nestmates via bee-to-bee contacts much as pollen is transferred among bees in the hive (DeGrandi-Hoffman et al. 1986). The rate that HBA is dispersed in colonies might be influenced by the effects of ambient temperature on clustering especially since HBA is a contact miticide. A higher percentage of bees might have HBA on their bodies when temperatures are low and bees are in cluster. During warmer periods such as those occurring during this study, better coverage might be achieved by re-positioning the strips among the frames. The best location of strips at different times of year and weather conditions should be explored further.

There was a decline in the percentage of bees with HBA in colonies after 7 days and a corresponding decrease in mite drop. Two weeks after application, HBA was no longer detected on bees. If the strips were still dispensing sufficient amounts of HBA, we would have detected it on bees and mite drop would have continued to some degree because mite infested brood was emerging. Indeed, when a second application was made in a subset of colonies, mite drop greatly increased 2 days later and resulted in a higher overall mite drop during the 21 day period. A similar decline in the effectiveness of HBA over time was found when HBA was applied in hop fields to control *T. uriticae* (Jones et al. 1996). If there is a loss of effectiveness 7 days after HBA is applied in honey bee colonies, a single application will not kill the mites that infested cells prior to the application. This is because developing workers are under the cell caps for 12 days and drones for 16 days (Winston 1987). The number of mites in sealed worker and drone brood can comprise a large portion of the overall mite population especially during the summer when colonies are in their peak brood rearing period (DeGrandi-Hoffman and Curry 2004). Therefore, a second or possibly a third application of HBA should be applied to kill the emerging mites especially in colonies with large brood areas.

After HBA strips were put in colonies, we inserted Apistan® strips to kill any remaining mites. We followed this with additional HBA strips and mite drop increased about two-fold. These findings indicate that there were fluvalinate resistant mites in the colonies. We established the colonies from packaged bees, and did not treat them with miticides prior to the HBA applications. If the packages had mites, this might have been the source of resistance. Fluvalinate is commonly found in the wax combs of hives (Mullin et al. 2010), therefore selection for resistance has continued even though Apistan® is rarely used. The HBA strips caused greater mite mortality than fluvalinate especially in the controls indicating that mites that are resistant to fluvalinate are susceptible to HBA.

A potentially important use of HBA is in package bees. Bee packages routinely contain some mites (Strange et al. 2008), and sometimes levels exceed the spring treatment threshold for certain regions of the US (Strange and Sheppard 2001). Colonies established with high varroa populations are unlikely to survive the year. The mites will infest the first brood reared in the colony and shorten the lives of emerged workers (De Jong and De Jong 1983; Schneider and Drescher 1987; Kovak and Crailsheim 1988) that are the replacements for the bees that comprised the package. The mite population will continue to grow with the colony throughout the spring and summer and by fall might be at levels that cause colony loss during the winter (Amden et al. 2004). In our study, more than 90 % of the mites in the packages were killed by HBA without queen loss and with negligible mortality to the bees. Only when 4 strips were used in 1.4 kg packages was worker mortality greater than in controls. Since the additional strip did not significantly increase mite mortality, no more than 3 strips should be used. Reducing mite populations in package bees can ultimately prevent colony losses by insuring that there are low numbers of mites when the colony is established. Varroa populations might be kept at low levels by subsequent applications of HBA or other miticides (e.g., formic acid) during broodless periods in the fall or winter.

HBA can affect feeding and ovipositional behavior of mites and cause repellency when applied to plants (Jones et al. 1996, 2003). We detected some repellency in our petri dish experiments where we applied HBA directly on to worker bees, and then exposed them to mites. However, our measurements in colonies and packages were exclusively of mortality. There might have been a reduction in feeding and oviposition behavior of the mites under capped cells that were exposed to sublethal levels of HBA during their phoretic stage. Future studies will be conducted to investigate this possibility. We could be underestimating the effectiveness of HBA if there is a decrease in reproductive success coupled with higher mite mortality.
